# Acute Abdominal Aorta Thrombosis and Ischemic Rhabdomyolysis Secondary to Severe Alcohol Intoxication

**DOI:** 10.7759/cureus.905

**Published:** 2016-12-01

**Authors:** Zain Ul Abideen, Syed Farhat Abbas, Madeeha Farooq, Amna Rasheed, Furqan Ali

**Affiliations:** 1 Nephrology and Renal Transplant, Shifa International Hospital, Islamabad, Pakistan; 2 Department of Nephrology, Shifa International Hospital, Islamabad, Pakistan; 3 Department of Internal Medicine, Shifa International Hospital, Islamabad, Pakistan

**Keywords:** rhabdomyolysis, alcohol intoxication, holiday heart syndrome, abdominal aorta thrombosis

## Abstract

Acute alcohol intoxication is a common cause of emergency visits worldwide. Although moderate alcohol consumption is protective against coronary artery disease, binge drinking is associated with adverse cardiovascular and neurological outcomes and may even cause sudden death. Although, few past accounts of venous thrombosis with alcohol binge drinking are available, arterial thrombosis with the condition has never been reported in the literature. We present the unusual case of a young Afghan male, who presented to us with painful, tender and swollen legs three days after a heavy alcohol binge on a Saturday night. He was diagnosed as a case of acute limb ischemia secondary to massive abdominal aorta and bilateral femoral artery thrombosis. He also had acute renal failure secondary to rhabdomyolysis. Cardiac workup revealed new onset paroxysmal atrial fibrillation and a large thrombus in the left ventricular cavity. His blood ethanol level was high. He was treated by a multidisciplinary team; urgent surgical thrombectomy for thrombotic complications, intravenous fluid hydration and later renal replacement therapy for acute renal failure. To the best of our knowledge, such a constellation of clinical features in association with severe acute alcohol intoxication has not been reported in the literature. We believe, the procoagulant nature of high blood ethanol levels and the onset of atrial fibrillation after the heavy alcohol binge, known as the holiday heart syndrome, precipitated the thrombotic events leading to rhabdomyolysis and acute renal failure. Through this case, we conclude that a very heavy alcohol binge may cause thrombotic occlusion of the abdominal aorta and femoral arteries resulting in ischemic rhabdomyolysis and acute renal failure. A high index of suspicion must be kept, especially for a patient presenting with tender, swollen lower limbs and acute renal failure after an alcohol binge.

## Introduction

Acute alcohol intoxication is a common cause of hospital emergency visits. It usually results from binge drinking, defined as the consumption of greater than five drinks on a single occasion [1-2]. A variety of manifestations may result after acute alcohol intoxication and these depend on the amount of alcohol consumed. They range from central nervous system manifestations like slurred speech, impaired cognition, unsteady gait and nystagmus, to acute hepatitis, hypotension, tachycardia, arrhythmias and life-threatening metabolic and electrolyte abnormalities. Though moderate alcohol use has beneficial effects on the body, excessive chronic alcohol abuse is implicated in liver disease, cardiomyopathy, and cancers of the mouth and esophagus [3]. Acute or binge drinking is associated with increased risk of sudden death [2]. A recent study also associated it with an increased risk of stroke [3].

The holiday heart syndrome was first described by Ettinger, et al. in 1978 after witnessing a peculiar disturbance of the heart rhythm in otherwise healthy patients who took an alcohol binge on weekends and holidays. Later it was revealed that the presence of weekends or holidays was not always the case. The condition improved after abstinence from alcohol. A variety of arrhythmias may be triggered, most commonly atrial fibrillation [3]. In addition, a few studies have found high blood alcohol levels to induce procoagulant changes, especially after binge drinking.

We describe the intriguing case of a young male who developed abdominal aorta and bilateral femoral artery thrombosis after a heavy alcohol binge. This culminated in ischemic rhabdomyolysis and acute renal failure. The patient also developed new-onset atrial fibrillation after the binge. We believe, the extensive thrombotic event was precipitated by the prothrombotic nature of high blood alcohol levels and atrial fibrillation. To the best of our knowledge, such a constellation of findings has never been reported as a complication of alcohol binge drinking. Informed consent was obtained from the patient for this study.

## Case presentation

A 30-year-old married male from Afghanistan presented with bilateral leg pain and swelling for the past one week. He also had worsening nausea, vomiting, and anorexia. The complaints started a few hours after he drank almost three bottles of 750 ml pure alcohol spirit at a Saturday night party and progressively worsened. He remained admitted to a hospital in Afghanistan and was being managed for acute renal failure. He was referred due to worsening lower limb swelling, pain, and renal functions. He had been drinking alcohol for the past four months on a regular basis. There was no other past medical or surgical history of note. On examination, his pulse was 139/minute, blood pressure 130/90 mm Hg, respiratory rate 14 per minute and he was afebrile. There was no edema. Examinations of the neurological, gastrointestinal and respiratory systems were unremarkable. A cardiovascular examination revealed weak femoral, absent popliteal and dorsalis pedis pulses. A lower limb examination revealed swollen, erythematous, warm and extremely tender lower limbs. The electrocardiogram (ECG) revealed new-onset atrial fibrillation with a rapid ventricular response(RVR).

The laboratory parameters are summarized in Table 1. Workup for autoimmune diseases and inherited and acquired thrombotic diseases was all negative.

**Table 1:** Laboratory parameters on admission.

Parameter	Patient value	Reference range
Hemoglobin	16.10 g/dL	13.5-18.5 g/dL
White cell count	12,000 /cumm	4-11000/cumm
Erythrocyte sedimentation rate (ESR)	85 mm/1^st^ Hour	Less than 20mm/1^st^ hour
Serum creatinine	7.82 mg/dL	Less than 1.2 mg/dL
Serum sodium	131 meq/L	135-145 meq/L
Serum potassium	5.9 meq/L	3.5-5.5 meq/L
Serum bicarbonate	10 meq/L	22-26 meq/L
Serum chloride	93meq/L	Up to 105 meq/L
Alanine amino transaminase (ALT)	426 U	Less than 40
Aspartate amino transaminase (AST)	401 U	Less than 40
Serum measured osmolality	316 mOsm/Kg	275-295 mOsm/Kg
Serum calculated osmolality	281 mOsm/Kg	
Osmolal gap	25 mOsm/Kg	Less than 10 mOsm /Kg
Serum urea	254 mg/dL	Less than 40 mg/dL
Creatine phosphokinase (CPK)	18777 U/L	Less than 200 U/L
Activated partial thromboplastin time (APTT)	20 seconds	21-34 seconds
Prothrombin time (PT)	11.5 seconds	11-13.5 seconds

**Figure 1:**
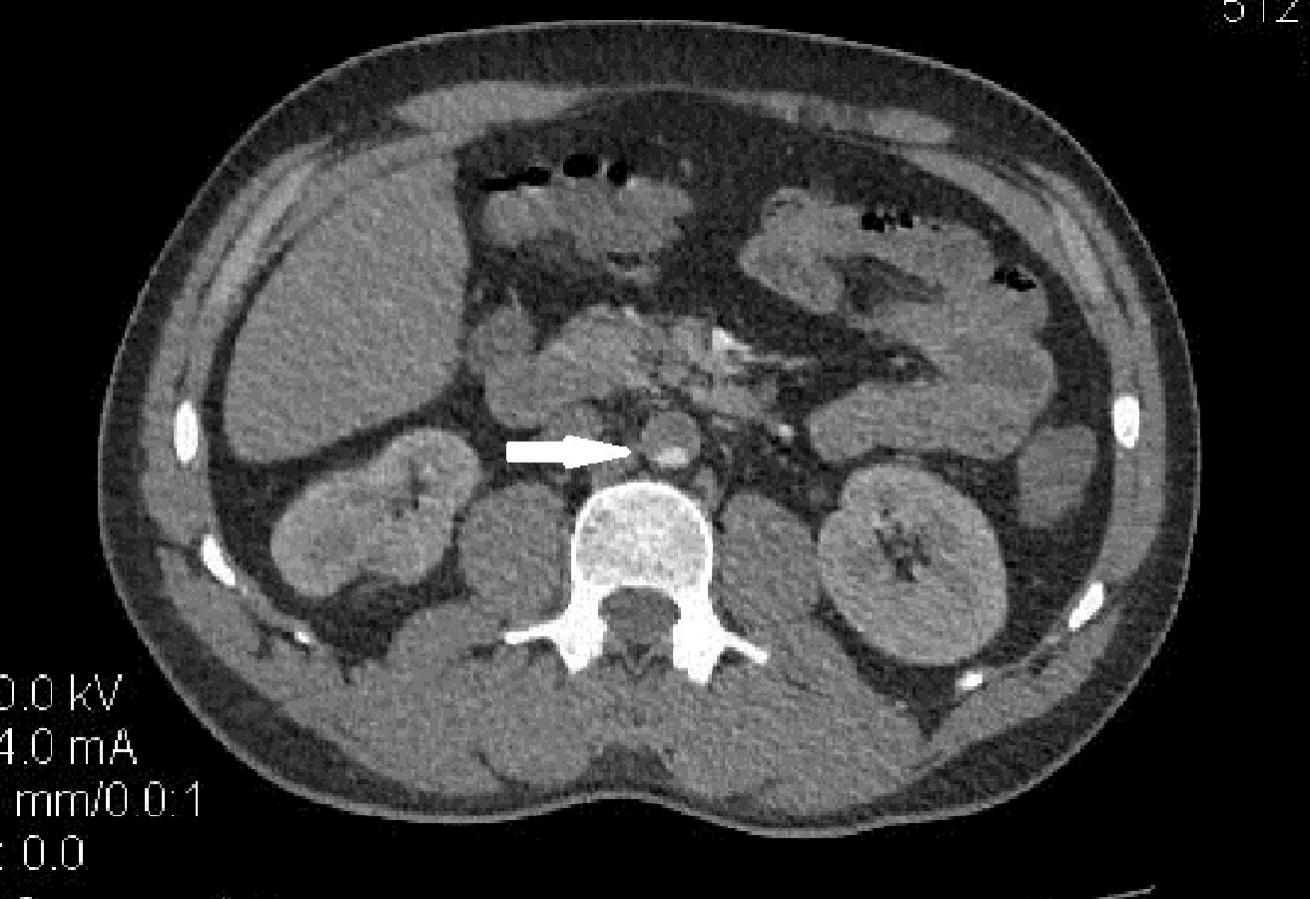
Computed tomography angiogram (CTA) of the abdomen. CTA shows a thrombosed abdominal aorta beyond origin of the renal arteries as indicated by the white arrowhead.

A Doppler ultrasound scan of the lower limbs revealed severe arterial insufficiency but no deep venous thrombosis. A computed tomography angiogram (CTA) revealed a thrombosed abdominal aorta beyond the origin of the renal arteries and femoral arteries were not visualized beyond the proximal thigh (Figure 1). A two-dimensional echocardiogram revealed a large intracardiac thrombus located in the left ventricular cavity.

He was scheduled for emergency surgery by the vascular surgery team. Bilateral femoral artery and aortic thrombectomy were successfully performed. The left side calf muscle was found to be dead and a part of it was removed. Good femoral and distal pulses were demonstrated by the Doppler ultrasound after the procedure. A muscle biopsy was also taken per-operatively, which showed inflammatory changes only. Warfarin was started at 7.5 mg once daily three days after the procedure. Heparin was started before the surgery once the thrombus was discovered and the dose was titrated to maintain the activated partial thromboplastin time (APTT) between 70 and 120 seconds.

Our patient underwent two sessions of hemodialysis on the first two days of his admission for worsening hyperkalemia and preparation prior to surgery. No ultrafiltrate was done. An intravenous fluid was continued throughout between 100-250 ml /hour as the patient was producing sufficient urine. The urine output was maintained persistently between 150-300 ml/ hour. Intravenous sodium bicarbonate was administered to maintain the urine pH above 6.5.

**Figure 2:**
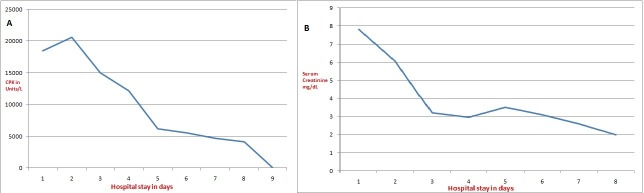
The trends of creatine phosphokinase (CPK) and serum creatinine during the hospital stay. Panel A- The trend of creatine phosphokinase (CPK) during the hospital stay. Panel B- The trend of serum creatinine during the hospital stay. Both parameters declined on initiation of treatment and thrombectomy.

The elevated liver enzymes (alcoholic hepatitis) was due to the alcohol binge. It was managed supportively and the enzymes normalized before discharge. He was discharged successfully two weeks after admission on warfarin 5 mg once daily. His international normalized ratio (INR) was maintained at 2.5. Two months after discharge a two-dimensional echocardiogram revealed resolution of the cardiac thrombus. The renal failure and alcoholic hepatitis had settled. Figure [2] shows the declining trend of serum creatinine and creatine phosphokinase (CPK) with treatment during the hospital stay.

## Discussion

Acute alcohol intoxication can have a constellation of signs and symptoms. Our patient had multiple manifestations of alcohol intoxication, which will be discussed in detail separately. The holiday heart syndrome (HHS) is the occurrence of cardiac arrhythmias, most commonly atrial fibrillation after alcohol binge drinking [1]. Binge drinking is defined as greater than five drinks taken at one time [2]. Binge drinking behavior is associated with an increased risk of sudden death and stroke; acute ingestion may cause spasm of the cerebral arteries while alcohol is itself associated with procoagulant changes after acute ingestion [3]. There is also an increased risk of sudden cardiac death with alcohol abuse and this rises with the amount of alcohol ingested. This may occur in those with no history of myocardial infarction or ischemic heart disease [1]. The syndrome is observed in both alcoholics and nonalcoholics and goes away on abstinence from alcohol. Important characteristics include the absence of any history or family history of any cardiac condition, normal laboratory parameters including electrolytes, thyroid hormones and echocardiogram. Our patient had a similar profile. Though an echocardiogram identified a left ventricular thrombus, there was no observation of any wall motion abnormality or any finding suggestive of a cardiomyopathy. The most common symptom reported by patients with HHS is palpitations. Our patient had this symptom for a week soon after binge drinking.

Although the observations of sudden death and stroke are well described with HHS, we could not find any account in the literature regarding HHS and acute abdominal aorta thrombosis and rhabdomyolysis. Abdominal aorta thrombosis is an uncommon clinical event. Its occurrence secondary to atrial fibrillation is even rarer with only a few case reports available on PubMed [4-5]. There is no account of the entity taking place after alcohol intoxication or HHS. Some associations described with abdominal aorta thrombosis include the presence of an aortic aneurysm, surgical manipulation, trauma, severe dehydration, hypercoagulable state, hypotension and neoplastic disease [5]. Our patient had none of these conditions. He was subjected to a thorough workup for any inherited thrombophilia, which turned out to be negative. The occurrence of a large thrombus in the left ventricular cavity and atrial fibrillation strongly indicated the source of the thrombus. However, such extensive thrombosis is even better explained by a procoagulant tendency of the blood. In our case, this was probably due to the excess alcohol levels in the blood.

It is well known that alcohol consumption in moderate quantities reduces the risk of coronary artery disease by activation of the fibrinolytic system, lowering platelet aggregation, antioxidant effects, lipid profile improvement including an increase in high-density lipoprotein levels, improved endothelial function and increasing insulin sensitivity [3-6]. However, acute ingestion of large amounts of alcohol can cause procoagulant tendencies in the blood by increasing thromboxane, factor VII and plasminogen activator inhibitor-1 (PAI-1) levels [7-8]. In addition, alcohol binge drinking predisposes to dehydration and hyperosmolality of the blood. There have been past reports of cerebral venous sinus thrombosis after binge drinking [9]. Our patient suffered acute severe alcohol intoxication. Though he presented to us after a week, on presentation to the initial health facility, he was drowsy and had slurred speech. Although blood ethanol levels do not correlate well with clinical signs and symptoms, levels between 200-300 mg/dL are usually associated with such a clinical picture. Our patient had a blood level of 130 and an osmolar gap of 25. Since he presented days after the binge, most of the alcohol had been metabolized.

Our patient also had nontraumatic ischemic rhabdomyolysis evidenced by the raised creatine phosphokinase levels. Alcohol may itself cause nontraumatic rhabdomyolysis, though this is a rare occurrence. The mechanism postulated is a disruption of the adenosine triphosphate (ATP) pump and skeletal membranes, induction of cytochrome p450 and alteration of sarcoplasmic reticulum [10]. Other mechanisms include prolonged coma, seizures, falls or immobility, and electrolyte disturbances; our patient had none of these [10]. Thus, it is likely that our case was complicated by ischemia and direct toxic effects of alcohol on the skeletal muscles. The rhabdomyolysis is treated by aggressive fluid hydration to maintain a urine output of 200-300 ml/hour in the first 24 hours. The urine pH should be kept above 6.5 to avoid precipitation of myoglobin in the renal tubules [10]. Although we were able to obtain the above objectives with intravenous normal saline and sodium bicarbonate respectively, the patient required two sessions of renal replacement therapy for worsening acidosis and hyperkalemia that had to be rectified before surgery for his aortic thrombus. He was monitored for electrolyte abnormalities and hypoglycemia. Acute abdominal and femoral artery thrombosis need urgent thrombectomy to avoid ischemia-induced tissue loss. Our patient benefited from the above procedure with resolution of the limb ischemia, rhabdomyolysis, and renal failure.

## Conclusions

Through this case, we conclude that a very heavy alcohol binge may cause thrombotic occlusion of the abdominal aorta and femoral arteries resulting in ischemic rhabdomyolysis and acute renal failure. The mechanisms may include the induction of a procoagulant state and cardiac arrhythmias like atrial fibrillation. A high index of suspicion must be kept, especially for a patient presenting with tender, swollen lower limbs and acute renal failure after an alcohol binge.
